# *In Situ* and *Ex Situ* X-ray Diffraction and Small-Angle X-ray Scattering Investigations of the Sol–Gel Synthesis of Fe_3_N and Fe_3_C

**DOI:** 10.1021/acs.inorgchem.1c03442

**Published:** 2022-04-26

**Authors:** Matthew S. Chambers, Robert D. Hunter, Martin J. Hollamby, Brian R. Pauw, Andrew J. Smith, Tim Snow, Ashleigh E. Danks, Zoe Schnepp

**Affiliations:** † School of Chemistry, University of Birmingham, Birmingham B152TT, U.K.; ‡ Department of Chemistry, School of Chemical and Physical Sciences, Keele University, Staffordshire ST55BG, U.K.; § Bundesanstalt für Materialforschung und -prüfung (BAM), Unter den Eichen 87, Berlin 12205, Germany; ∥ Diamond Light Source, Didcot, Oxfordshire OX11 0DE, England

## Abstract

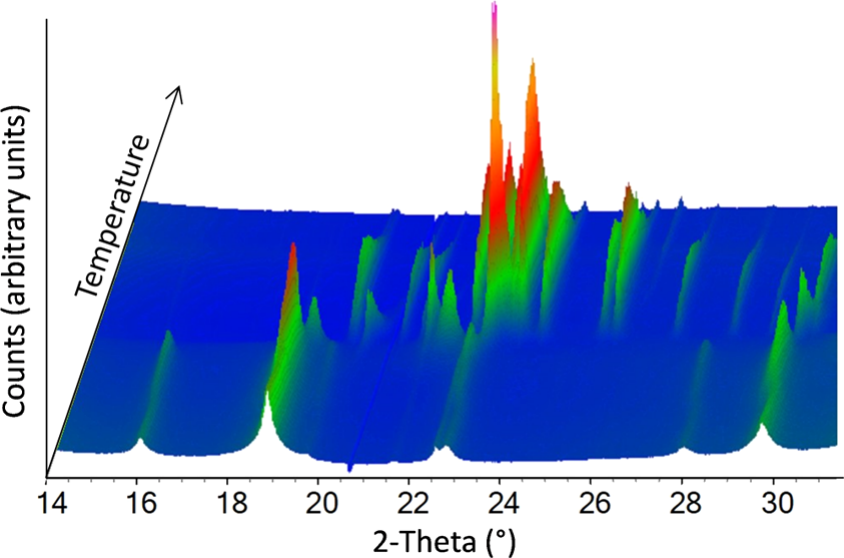

Iron nitride (Fe_3_N) and iron carbide (Fe_3_C) nanoparticles can be prepared via sol–gel synthesis. While sol–gel methods are simple, it can be difficult to control the crystalline composition, *i.e.*, to achieve a Rietveld-pure product. In a previous *in situ* synchrotron study of the sol–gel synthesis of Fe_3_N/Fe_3_C, we showed that the reaction proceeds as follows: Fe_3_O_4_ → FeO*_x_* → Fe_3_N → Fe_3_C. There was considerable overlap between the different phases, but we were unable to ascertain whether this was due to the experimental setup (side-on heating of a quartz capillary which could lead to thermal gradients) or whether individual particle reactions proceed at different rates. In this paper, we use *in situ* wide- and small-angle X-ray scattering (wide-angle X-ray scattering (WAXS) and small-angle X-ray scattering (SAXS)) to demonstrate that the overlapping phases are indeed due to variable reaction rates. While the initial oxide nanoparticles have a small range of diameters, the size range expands considerably and very rapidly during the oxide–nitride transition. This has implications for the isolation of Rietveld-pure Fe_3_N, and in an extensive laboratory study, we were indeed unable to isolate phase-pure Fe_3_N. However, we made the surprising discovery that Rietveld-pure Fe_3_C nanoparticles can be produced at 500 °C with a sufficient furnace dwell time. This is considerably lower than the previous reports of the sol–gel synthesis of Fe_3_C nanoparticles.

## Abstract 2

*In situ* synchrotron small- and wide-angle X-ray scattering have been used to probe the sol−gel synthesis of Fe_3_C, showing how particle size evolves during the various intermediate crystalline transformations. Experimental investigations also show that while it is difficult to isolate phase-pure Fe_3_N, it is possible to produce Fe_3_C at the surprisingly low temperature of 500 °C.

## 1 Introduction

**Figure 1:**
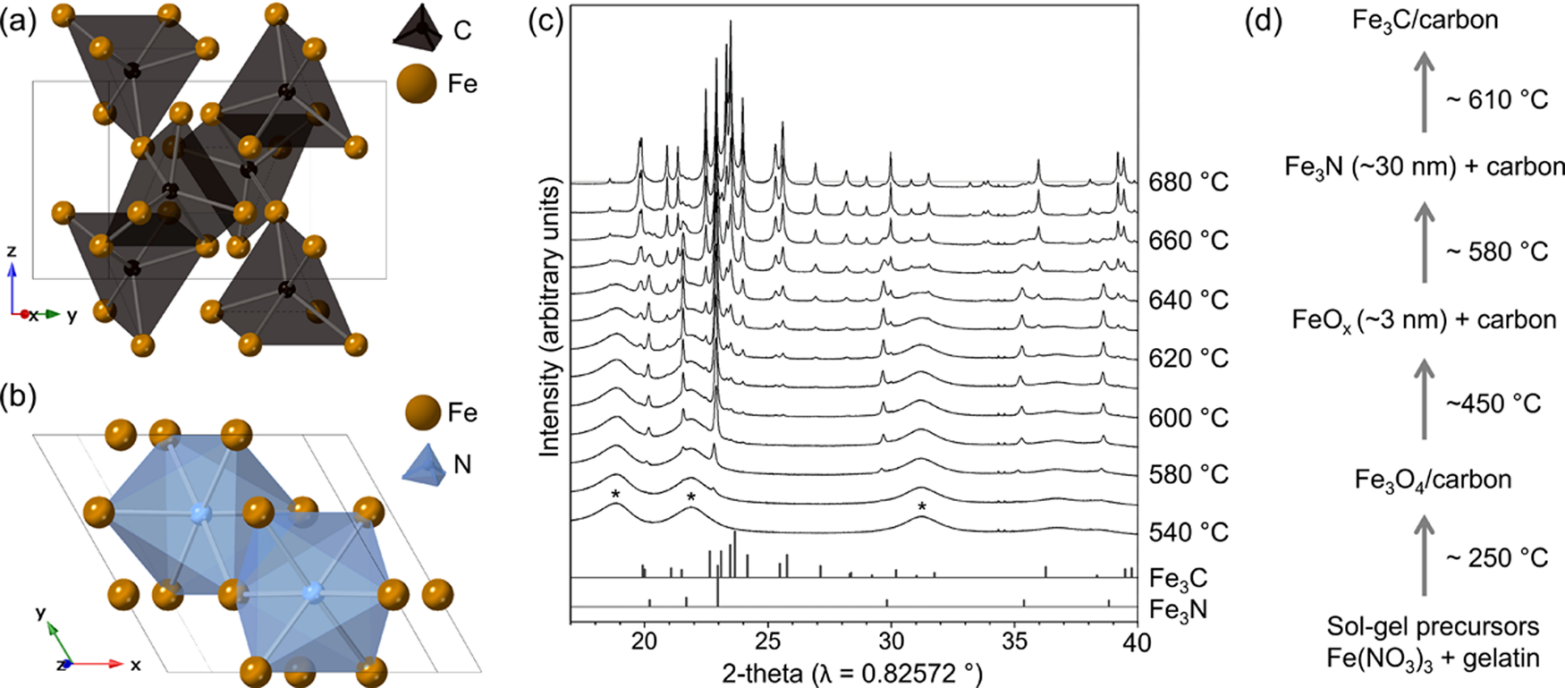
Crystal structures of (a) θ-Fe_3_C and (b) ε-Fe_3_N.^18^ (c) *In situ* synchrotron X-ray diffraction data showing part of the mechanism of formation of Fe_3_C from a gelatin/iron nitrate precursor^16^ and (d) schematic showing the approximate onset of each phase transition. Peaks marked with * correspond to FeO*_x_* (Wüstite). Images modified with permission.

Interstitial iron compounds θ-Fe_3_C and ε-Fe_3_N (Figure 1a,b) have recently gained attention due to their potential applications as nanoparticle catalysts in the oxygen reduction reaction,^1^ the Fischer–Tropsch process,^2,3^ and ammonia decomposition.^4^ They are particularly appealing due to their potential to replace precious metals such as Pt in these processes.^5,6^ Furthermore, Fe_3_C and Fe_3_N nanoparticles possess interesting magnetic properties and could be used in biomedical applications.^7−9^ There are various synthetic routes to produce Fe_3_N and Fe_3_C nanoparticles, including laser ablation,^10^ ammonolysis,^11^ solvothermal synthesis,^8,12^ nanocasting,^13^ and sol–gel synthesis.^14−16^ Sol–gel chemistry is particularly promising due to its simplicity both in reactants and technical implementation. Sol–gel synthesis uses gel or gel-like mixtures of metal and organic species as precursors for ceramic materials.^17^ Heating the gel leads to nucleation and growth of ceramic material (*e.g.*, metal oxides, nitrides, and carbides). For example, Fe_3_C can be produced by heating a mixture of Fe(NO_3_)_3_ and gelatin to 700 °C in nitrogen.

To understand how Fe_3_C is formed in sol–gel synthesis, we previously reported an *in situ* synchrotron X-ray powder diffraction study.^16^ The data showed that thermal decomposition of Fe(NO_3_)_3_/gelatin proceeds through several intermediates, including Fe_3_O_4_ and FeO_*x*_ (Figure 1c,d). The iron oxide peaks were very broad, indicating small crystallite sizes (mean diameter estimated as 3 nm by Scherrer equation). The broad iron oxide peaks were gradually replaced with sharp Fe_3_N peaks from 560 °C and Fe_3_C peaks from 610 °C. This indicated that the nitride and carbide phases comprised much larger crystalline domains (estimated 30 and 60 nm diameters, respectively). Peak shifts suggested that the Fe_3_N to Fe_3_C phase transition was caused by carbon diffusion via a carbonitride intermediate.

Recently, we reported a further *in situ* synchrotron investigation of the Fe(NO_3_)_3_ system using total scattering.^18^ This revealed that there is a dramatic increase in order within the iron oxide nanoparticles between 300 and 350 °C. This was indicated by a transition from no correlations in the pair distribution function (PDF) at *r* > 6 Å at the lower temperature to correlations up to 40 Å at 350 °C. This could result either from sintering of very small crystallites or a fast crystallization of amorphous FeO*_x_* clusters. Additionally, small-box PDF refinements revealed the presence of locally distorted NFe_6_ octahedra within Fe_3_N, with a twist angle^19,20^ of φ = 49.51(1)°; more trigonal prismatic in nature than the high-symmetry, long-range twist angle of φ = 57.96°. As Fe_3_C contains CFe_6_ trigonal prisms, this is potentially further evidence that carbon is doped into the Fe_3_N lattice, replacing N atoms.

The previous experiments offered many insights into the sol–gel synthesis of Fe_3_C, but several questions remain. The first is how the particle size evolves over the whole reaction. Analysis of powder diffraction data using the Scherrer equation was used to give an estimate of the mean particle size for each phase but could give no information on particle size distribution. Furthermore, the *in situ* diffraction data showed significant overlap between the different phases, with FeO*_x_*, Fe_3_N, and Fe_3_C all coexisting between 610 and 660 °C. It is unclear whether this is a factor inherent to the system (*i.e.*, some crystallites are reduced more readily than others) or whether it is a feature of the *in situ* synchrotron experimental setup where a hot air blower was used to heat the sample within a quartz capillary, potentially resulting in a thermal gradient across the sample. Therefore, we have performed an *in situ* synchrotron study using a quartz capillary inside a modified tube furnace to provide a more consistent heating rate. We recorded small-angle X-ray scattering (SAXS) and wide-angle X-ray scattering (WAXS) simultaneously so that particle size distribution could be coupled to the evolution of the different crystalline phases. We then report a detailed *ex situ* experimental study of this system to probe the effect of heating conditions on the stability of the various phases. These papers together give a complete and rigorous picture of the Fe(NO_3_)_3_/gelatin sol–gel system across multiple length scales.

## 2 Experimental Procedure

### 2.1 Synthesis

For all samples, the gelatin precursor was prepared as discussed in previous literature.^16^ Briefly, a hot aqueous gelatin solution (10% w/w, 10 g; Sigma-Aldrich, G2500) was mixed with aqueous iron nitrate (10% w/v, 20.2 mL, Fe(NO_3_)_3_·9H_2_O, Sigma-Aldrich), forming a viscous orange gel. The gel was dried in air at 70 °C to form a brittle orange-brown foam. For the samples studied with powder diffraction *ex situ*, the brittle foam samples were ground with a mortar and pestle and were heated under N_2_ atmosphere with a heating rate of 5, 7.5, or 10 °C^–1^ to various final temperatures and with various dwell times, which are discussed below. For SAXS–WAXS experiments, the orange foam was preheated at 250 °C under nitrogen in a muffle furnace to remove water and avoid expansion of the sample within the capillary during the experiment.

### 2.2 *Ex Situ* Powder Diffraction

*Ex situ* powder diffraction was performed on a Bruker D2 PHASER using an approximate 2:1 mixture of Co Kα_1_ (λ = 1.7899 Å) and Kα_2_ (λ = 1.7929 Å) radiation, Ni filter, and LYNXEYE detector. Samples were mounted on Si zero-background slides and scanned over a range of 10° ≤ 2θ ≤ 80°.

### 2.3 SAXS–WAXS

**Figure 2:**
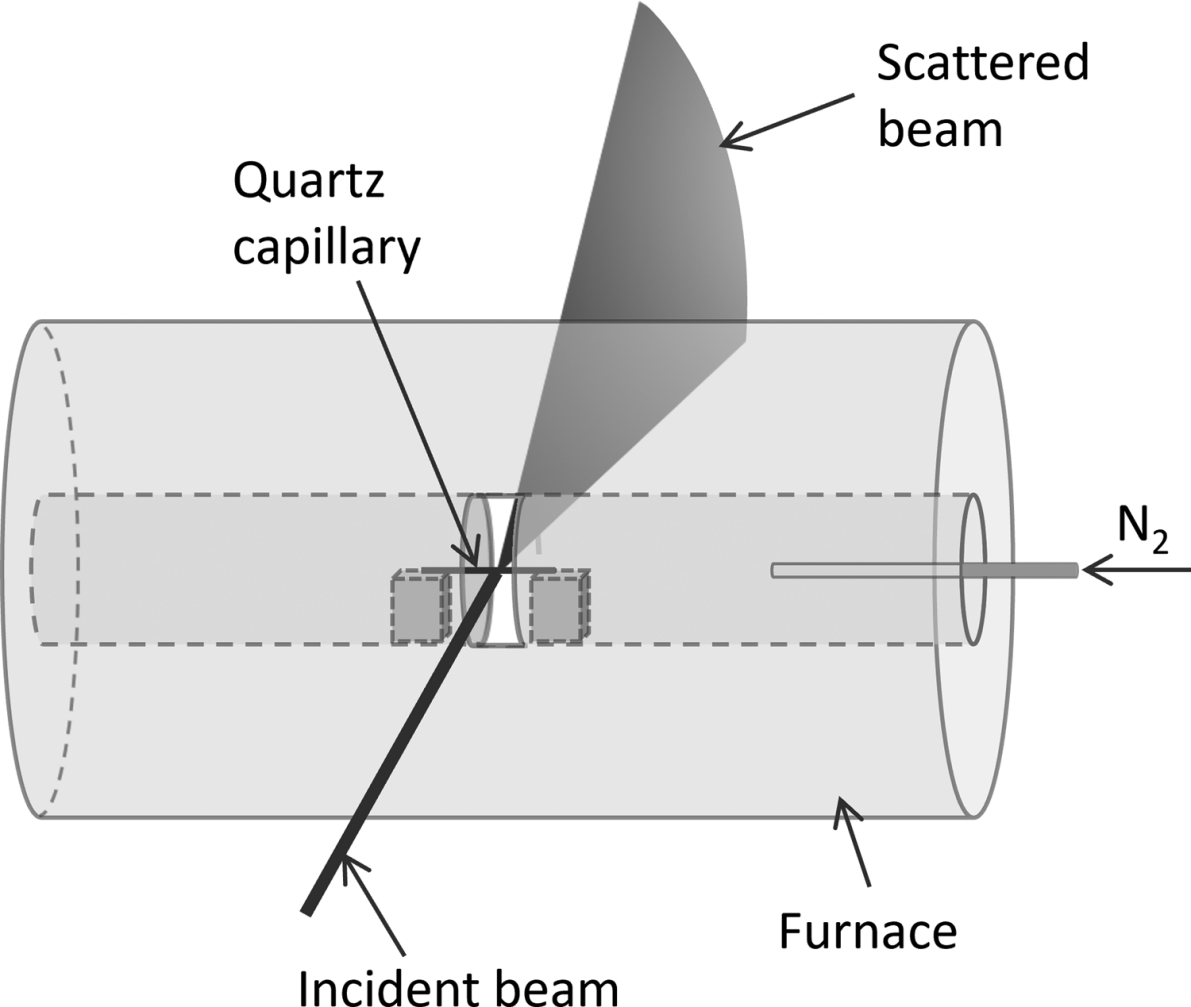
Schematic of the experimental setup (not to scale).

For SAXS/WAXS experiments, a preheated gelatin/Fe(NO_3_)_3_ sample was ground to powder and loaded into a quartz capillary (0.7 mm diameter, 0.02 mm wall thickness) and packed on either side with quartz wool to prevent the movement of the powder during heating. Measurements were performed on the I22 beamline at Diamond Light Source, using a beam energy of 15 keV (wavelength = 0.8266 Å), a sample to detector distance of 2.730 m, and a beam size of 200 μm × 180 μm. The scattered X-rays were detected using a Pilatus P3-2M unit from Dectris, which has a pixel size of 172 μm × 172 μm. The capillary was heated inside a modified tube furnace (Figure 2) with small slits cut in both sides for the incident and scattered beam, and nitrogen gas was flowed around the capillary during heating. Full details on the data correction and analysis procedures can be found in the Supporting Information.

### 2.4 Rietveld Refinement

Rietveld refinements were performed using TOPAS v6.^21,22^ Starting models for four phases were derived from the following sources: Fe_3_C from Wood et al.,^23^ Fe_3_N from Jacobs et al.,^24^ FeO_*x*_ (refined with a fixed stoichiometry of FeO) from Fjellvåg et al.,^25^ and Fe_3_O_4_ from Fleet^26^ (refined as fixed stoichiometry Fe_3_O_4_). Backgrounds were refined as twelfth-order Chebyshev polynomials. Peak shapes were described using a Thompson-Cox-Hastings pseudo-Voight function. Additionally, a zero-point error was refined. In the *ex situ* refinements, a strain-size line-broadening function^27^ was included for Fe_3_O_4_ at low temperatures, refining both the size and strain components, while for the WAXS refinements, two independent functions were included for FeO*_x_* and Fe_3_N. WAXS data were converted from *Q* to 2θ using an in-house Python script, given that . This was done as the Thompson-Hastings-Cox peak shape is defined in units of 2θ. To determine which phases were present in the WAXS refinements, preliminary refinements were performed with all four phases; phases found to not be present were then eliminated from certain temperature ranges and scan numbers. To study the thermal evolution of the Fe_3_N and Fe_3_C cell parameters, parametric refinements^28^ were performed against the data in the temperature range of 675–800 °C (first scan at this temperature), where Fe_3_N and Fe_3_C are the only crystalline phases present.

## 3 Results and Discussion

### 3.1 *In Situ* SAXS–WAXS Experiment

**Figure 3:**
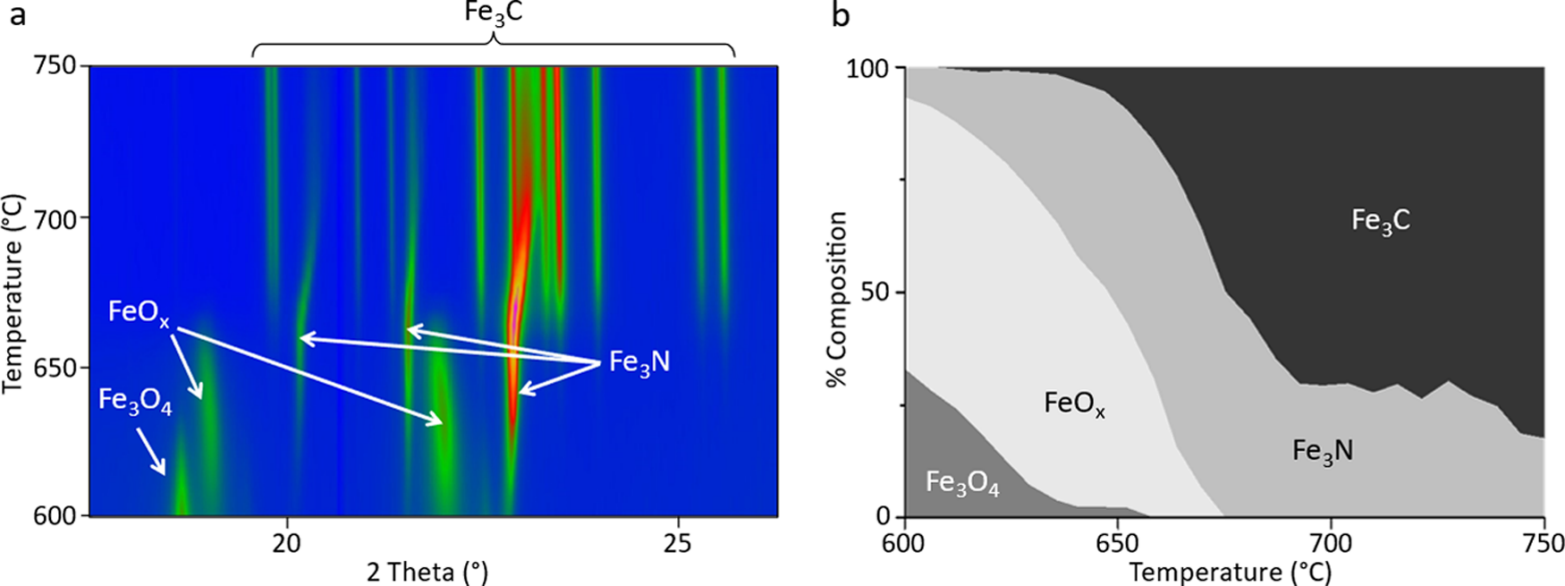
(a) Surface plot of *in situ* synchrotron WAXS data, where the colors represent an increase in peak intensity from blue to green to pink and (b) graph of % phase composition (from Rietveld refinement) during heating of the sample.

A sample of Fe(NO_3_)_3_/gelatin was preheated to 250 °C in nitrogen and then loaded to a quartz capillary and heated under a nitrogen atmosphere in a modified tube furnace. Synchrotron WAXS and SAXS data were collected throughout, and then Rietveld refinements were performed against 200 scans (78 of which are at 800 °C), starting from the same initial models for each phase. Figure 3a shows a surface plot of the WAXS data within the 600–750 °C region, where most of the phase transformations occur and the data clearly illustrate the Fe_3_O_4_–FeO*_x_*–Fe_3_N–Fe_3_C sequence. Phase compositions (from Rietveld refinement) are shown in Figure 3b and indicate the gradual transformation of the oxide phases to nitride and then carbide. To learn more from this data, it is useful to examine different regions of the heating process in turn.

**Figure 4:**
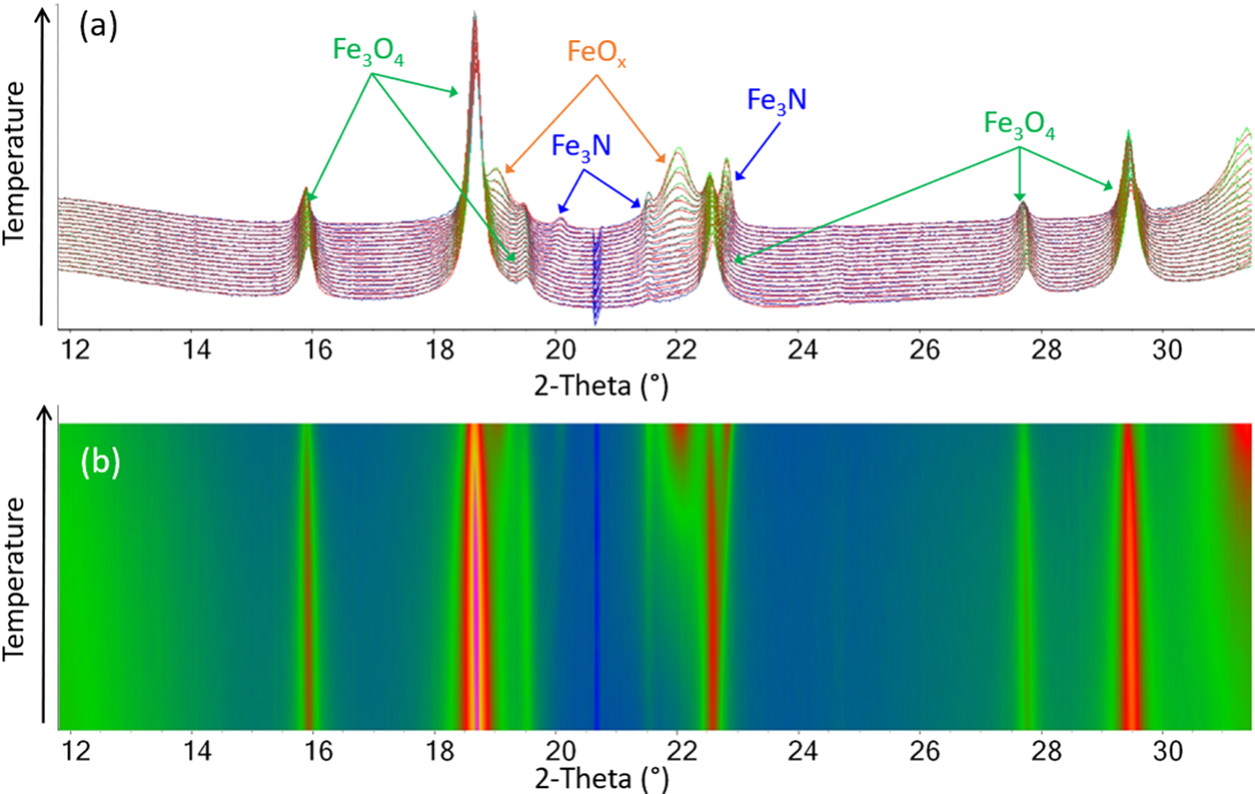
(a) Rietveld plots obtained from the WAXS data for temperatures 500–600 °C, vertically stacked along the *y*-axis, where the colors represent an increase in peak intensity from blue to green to pink and the orange line is the calculated pattern. The difference between the calculated and observed pattern is shown in Figure S1. (b) Surface plot of the same data, where the colors represent the same peak intensity increase.

Figure 4 shows the Rietveld plots obtained for the temperatures 500–600 °C. In this range, Fe_3_O_4_ is gradually reduced to FeO*_x_*. The peaks from FeO*_x_* are very broad, which could be caused by the small size of the particles and/or the highly disordered nature of FeO*_x_*.^25^ Fe_3_N begins to make a minor but noticeable contribution to the patterns at ∼530 °C, where the (21̅0) and (002) peaks are visible at 2θ ≈ 19.9 and 21.5°, respectively. Due to the peak broadening function used in these refinements,^27^ it is difficult to obtain precise compositions, but throughout this entire temperature range, the iron oxides dominate the composition. The gap in the diffraction data between 20 and 21° is related to the detector.

**Figure 5:**
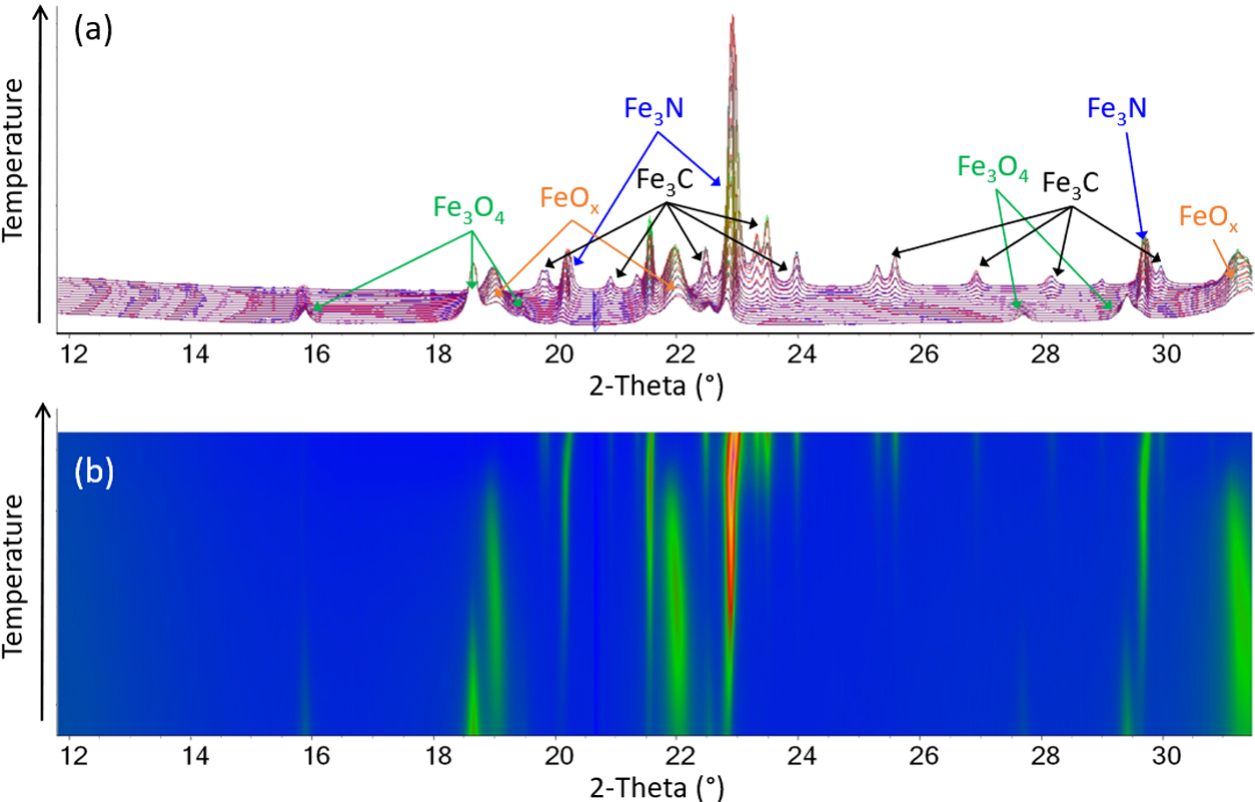
(a) Rietveld plots obtained from the WAXS data for temperatures 600–675 °C, vertically stacked along the *y*-axis, where the colors represent an increase in peak intensity from blue to green to pink and the orange line is the calculated pattern. The difference between the calculated and observed pattern is shown in Figure S1. (b) Surface plot of the same data, where the colors represent the same peak intensity increase.

**Figure 6:**
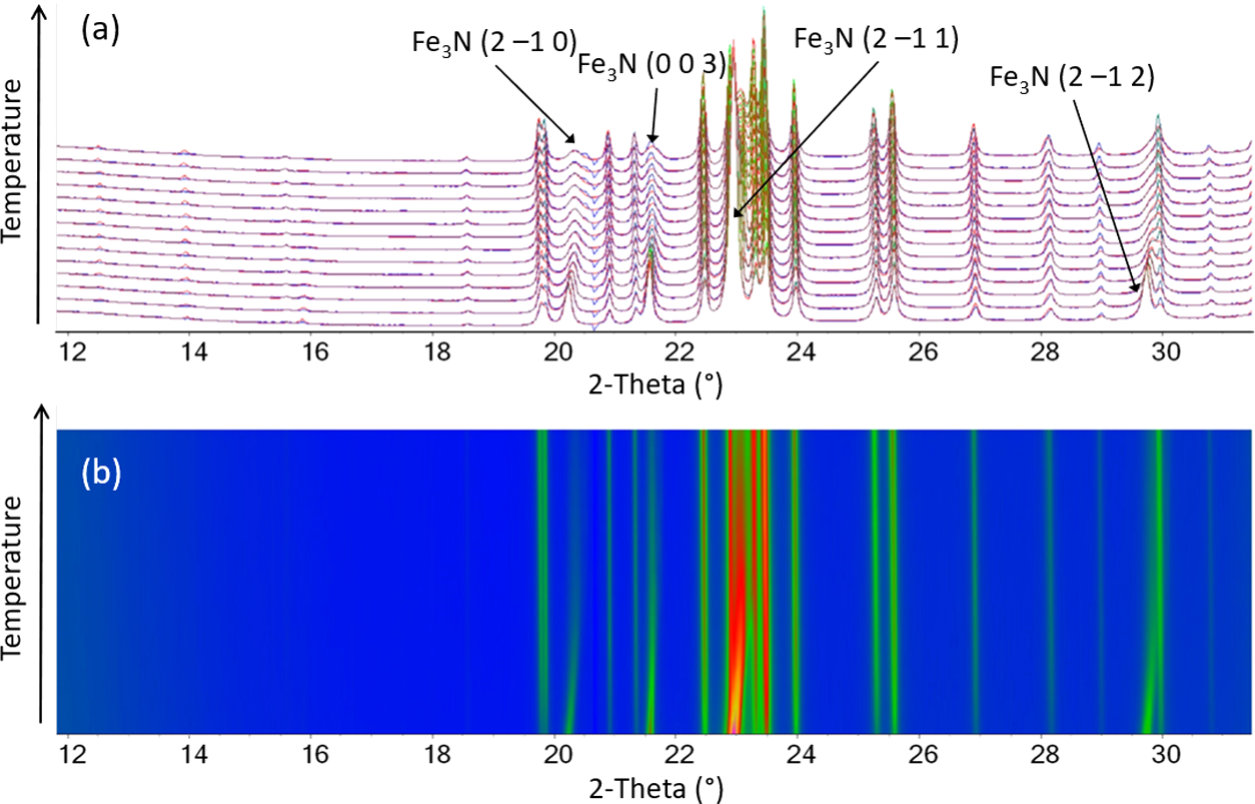
(a) Rietveld plots obtained from the WAXS data for temperatures 675–750 °C, vertically stacked along the *y*-axis, where the colors represent an increase in peak intensity from blue to green to pink and the orange line is the calculated pattern. The difference between the calculated and observed pattern is shown in Figure S1. (b) Surface plot of the same data, where the colors represent the same peak intensity increase.

**Figure 7:**
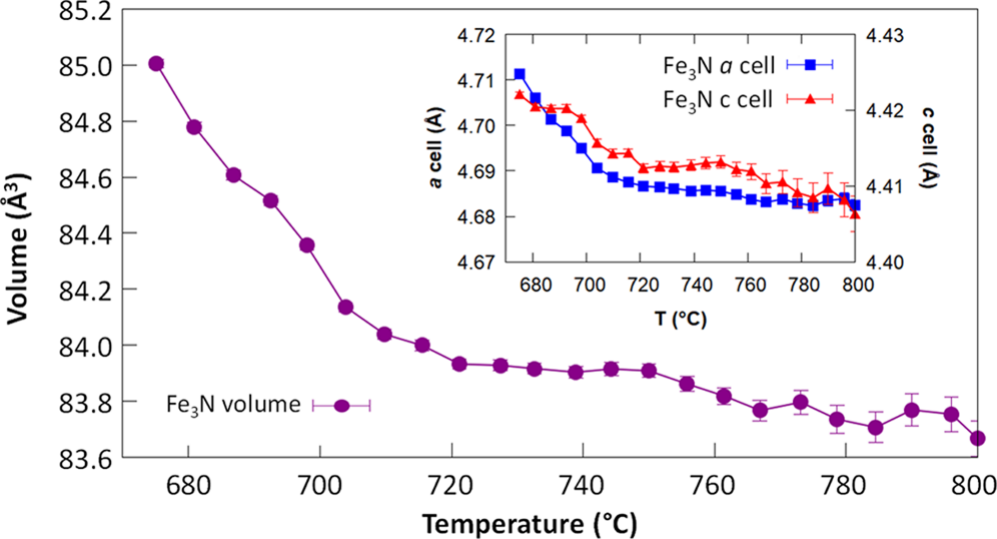
Thermal evolution of the cell parameters of Fe_3_N in the temperature range of 675–800 °C.

Figure 5 shows the Rietveld plots obtained for the temperatures 600–675 °C. In this range, the Fe_3_O_4_ is completely consumed, alongside an increase in the intensity of the FeO*_x_* peaks. Fe_3_N emerges alongside FeO*_x_*, and Fe_3_C forms toward the end of this temperature region, alongside the disappearance of the FeO*_x_* phase. By 675 °C, the only two (crystalline) phases present are Fe_3_N and Fe_3_C. The Rietveld plots for the 675–750 °C range are given in Figure 6. In a similar manner to the FeO*_x_* peaks, the Fe_3_N peaks first increase in intensity and sharpen with increasing temperature, indicating the growth of the particles and/or an increase in crystallinity. However, as Fe_3_C emerges, the Fe_3_N peaks broaden significantly and then disappear. A shift to higher 2θ can be observed from 675 °C in the Fe_3_N peaks, which is particularly noticeable in the (21̅0), (003), (21̅1), and (21̅2) peaks. This is most apparent in the surface plot shown in Figure 6b. This indicates a decrease in the cell parameters rather than the expected increase from thermal expansion. For a more quantitative analysis, parametric variable-temperature refinements were performed in the temperature range of 675–800 °C. Parametric refinements were performed as the same two phases (Fe_3_N and Fe_3_C) are present in this temperature range, and this allows more accurate cell parameters to be obtained. The results for Fe_3_N cell parameters are shown in Figure 7.

It is difficult to obtain accurate cell parameters in this system due to the broadening of the peaks, the overlap of Fe_3_C and Fe_3_N peaks, and the interference of the detector issue at 2θ = 20.7° (which affects the (21̅0) peak at the higher temperatures). However, there is a clear trend between 675 and 710 °C of the peaks shifting to a higher 2θ, which indicates a contraction of the lattice. Lattice contractions in Fe_3_N can be ascribed to loss of nitrogen,^29^ as iron nitride can be nonstoichiometric (Fe*_x_*N). Alternatively, the substitution of nitrogen atoms with carbon atoms in the formation of a ternary carbonitride phase has also been shown to result in a lattice contraction.^30^ Given the excess of carbon that is present in our system, it seems more likely that this data shows the existence of an iron carbonitride intermediate. This would also fit with our previous observations of local distortions of the Fe_6_N octahedra to a more trigonal pyramidal character during the Fe_3_N to Fe_3_C transition.^18^

**Figure 8:**
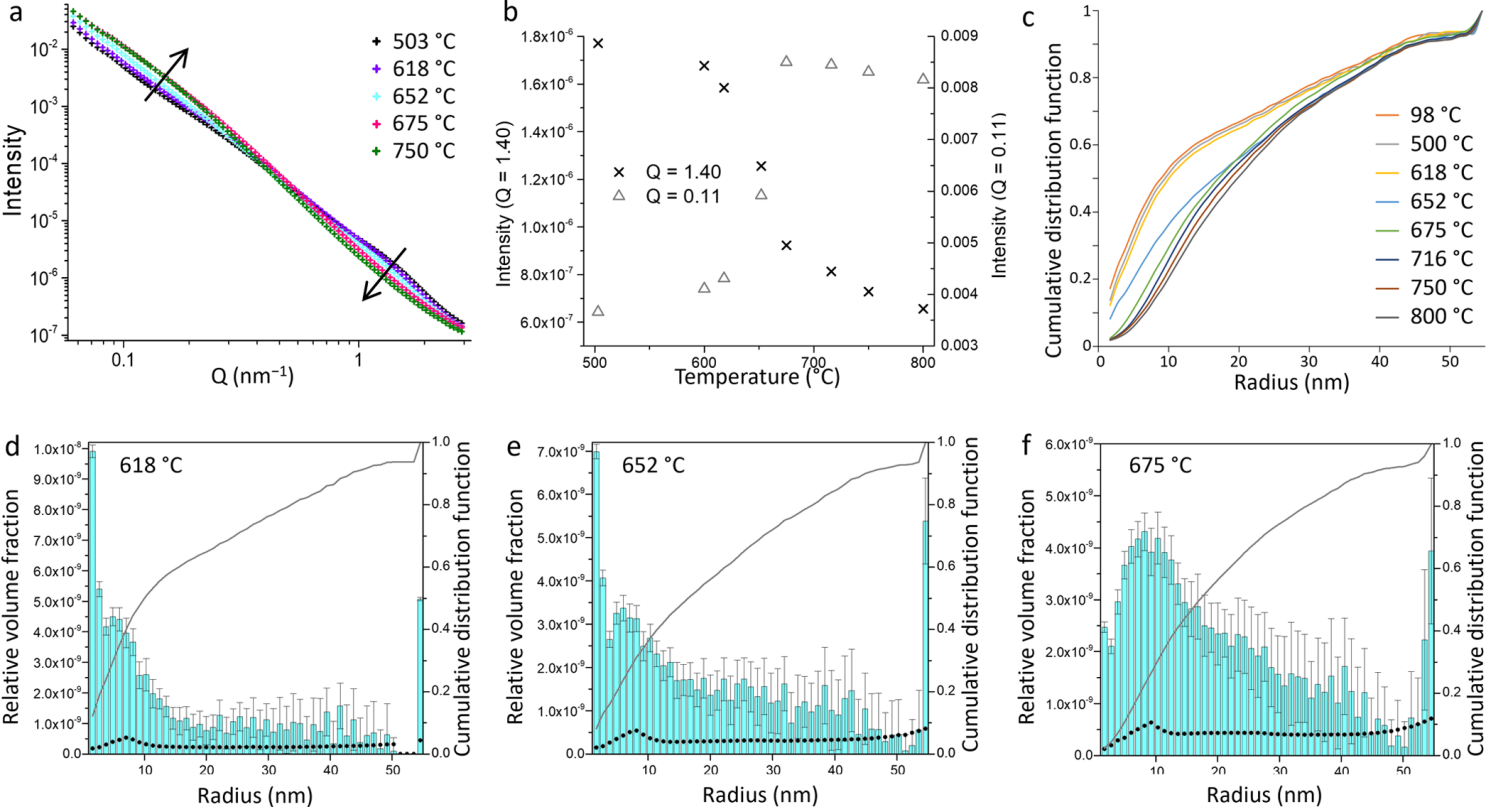
Graphs of (a) SAXS data at a selection of temperatures, (b) plots of intensity vs temperature at *Q* = 1.40 and *Q* = 0.11, and (c) cumulative distribution functions of particle radius for a range of temperatures. Particle size histograms derived from the SAXS data at (d) 618 °C, (e) 652 °C, and (f) 675 °C, with blue bars showing relative volume fraction, black dots showing minimum visibility limit, and gray lines showing the cumulative distribution function.

SAXS data were collected at the same time as the WAXS data to examine the evolution of the particle sizes. The SAXS data at a selection of temperatures (chosen to cover the regions of the phase transformations) are shown in Figure 8a, and there is clearly a gradual decrease in intensity at high *Q* alongside an increase in intensity at low *Q*. The change is not large but indicates an increase in the number of larger scattering features within the sample, as the temperature increases. The shift in intensity is not uniform. This is clearly shown in plots of intensity vs temperature at *Q* = 1.40 and 0.11 (Figure 8b). These plots show that the change in scattering intensity occurs over a short temperature range between ∼618 and 675 °C. This is concurrent with the oxide-to-nitride transformation and is consistent with the observation of sharper Bragg diffraction peaks for the nitride phase.

The SAXS data were analyzed using the Monte Carlo method^31^ to extract form-free size distributions. The data and corresponding fit lines for a selection of temperatures are shown in the Supporting Information (Figures S2–S10). We chose not to perform fits across all of the data sets, as this was unlikely to add any insight into the system. Radius histograms for the selected samples, scaled by relative volume fraction, are shown in Figures 8 and S11. From 98–600 °C, there is very little change in the particle size distribution, with approximately 50% of the particles having a radius <10 nm. In our previous investigation of this system using total scattering, we observed a dramatic increase in correlations >10 Å between 300 and 350 °C.^18^ The suggested conclusion from that study was that poorly crystalline regions of iron oxide were undergoing a rapid crystallization step rather than sintering of smaller crystals. The consistency in the particle size distributions from SAXS across this temperature range adds further weight to that argument. This is because SAXS would distinguish between small and large crystallites, whereas no change in scattering intensity would be shown for a transition between a disordered iron oxide cluster and a crystal of the same size. The particle size distributions from SAXS data show a shift to larger particles between 618 and 675 °C, which correlates with the emergence of Fe_3_N peaks in the WAXS data. This is consistent with much sharper diffraction peaks for the iron nitride phase and further supports the conclusion that the iron nitride particles are larger than the iron oxide precursor particles. What is surprising is the rate at which the particle size increases. This can be seen more clearly in a plot of the cumulative distribution functions (Figure 8c), where there is a sudden jump to higher particle radii between 618 and 652 °C. This strongly indicates that the iron oxide-to-nitride transition involves mass transport of iron through the carbon matrix rather than direct nitridation of individual oxide particles, a mechanism that was proposed but not proven in our previous work.^16^ Overall, the particle size distribution during the Fe_3_N and Fe_3_C evolution is much broader than is observed when the system contains only FeO*_x_*. Given that the distributions will contain contributions from the iron oxide, nitride, and carbide phases, it is perhaps not surprising that they are broad. Smaller nanoparticles are likely to react faster, and Fe_3_C nanoparticles that form early in the synthesis will have more time to sinter and grow. However, sol–gel chemistry is well known for producing particles that are relatively similar in size due to the homogeneous nature of the starting material. In this system, the rapid growth during the oxide-to-nitride transition indicates that the iron species are highly mobile, resulting in polydispersity despite a relatively narrow particle size distribution in the oxide precursor.

### 3.2 *Ex Situ* Experimental Study

**Figure 9:**
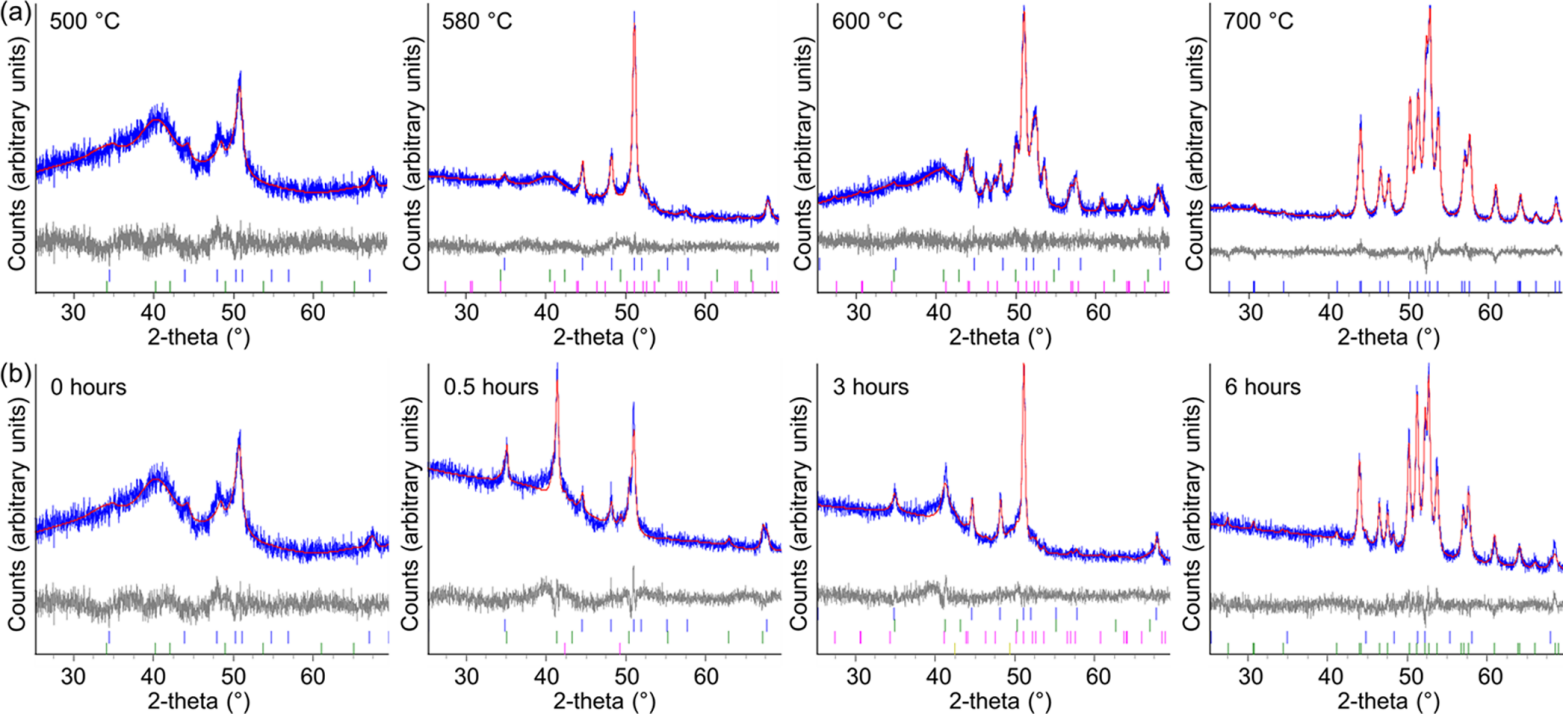
XRD data (Co Kα) and Rietveld plots obtained *ex situ* from the sol–gel synthesis of Fe(NO_3_)_3_/gelatin system with a heat rate of 5 °C min^–1^ and (a) 0 h dwell time at various temperatures and (b) different dwell times at a maximum temperature of 500 °C. The blue curves represent the observed data; the red curves, the calculated pattern; and the gray curves, the difference between the two patterns. The tick marks represent different reference peaks, including Fe_3_O_4_ (green), FeO*_x_* (orange), Fe_3_N (blue), and Fe_3_C (pink).

*In situ* SAXS/WAXS data showed progressive transitions from oxide–nitride–carbide with considerable overlap between the three phases. However, since the system was heated continuously, it did not allow for the stabilization of the system at the nitride stage. To investigate whether it is possible to produce phase-pure iron nitride in a laboratory furnace by this sol–gel method, we conducted an extensive *ex situ* experimental study. Figure 9a shows diffraction patterns of Fe(NO_3_)_3_/gelatin samples heated at 5 °C min^–1^ to various temperatures with no dwell time at the maximum temperature. Composition data from Rietveld refinement can be found in Table 1. The poor crystallinity of the samples and low signal-to-noise ratio means that the composition values have large errors, but some useful trends can still be identified. At 500 °C, the XRD pattern is noisy and shows broad peaks for Fe_3_N (40%) and Fe_3_O_4_ (60%). Heating up to 560 °C results in a similar composition (41% Fe_3_N, 59% Fe_3_O_4_), though with slightly sharper peaks, as might be expected from increased crystallization at the higher temperature (Figure S12). At 580 °C, most of the sample is Fe_3_N (70%), but Fe_3_C appears in small quantities (7%), and the sample still contains Fe_3_O_4_ (23%). At 600 °C, Fe_3_C is the dominant phase (58%), yet Fe_3_O_4_ and Fe_3_N remain in the sample. By 700 °C, the sample is 100% Fe_3_C, with relatively sharp peaks, indicating higher crystallinity. These data show that it is not possible to isolate a pure iron nitride phase with a fast reaction time, which reflects the results from the *in situ* study.

**Table 1:** Weight Percentage Compositions for Samples Heated at 5 °C min^–1^ to Various Temperatures with 0 h Dwell Time. Errors are Shown in Brackets

	Fe_3_O_4_	Fe_3_N	Fe_3_C
500 °C	60(18)	40(18)	
560 °C	41(12)	59(12)	
580 °C	23(7)	70(7)	7(2)
600 °C	25(8)	16(2)	58(6)
650 °C			100
700 °C			100

To try and isolate the intermediate iron nitride phase, we heated samples of the iron nitrate/gelatin precursor to various temperatures with a dwell time of 0.5 or 1 h. The composition data for both series show the same general trend (Tables S1 and S2), where iron oxide is gradually converted to iron nitride and then carbide. The samples held at 500 °C for 0.5 and 1 h show sharper diffraction peaks for iron oxide than the sample with no dwell time, which is consistent with crystallinity increasing with the longer sintering time. However, the sharp iron oxide peaks at 500 °C give way to broader iron oxide peaks at 520 °C (Figure S13), suggesting that the iron oxide crystallites become smaller and more disordered as they are consumed during carbothermal reduction. Another observation from these data is that complete formation of Fe_3_C happens at lower temperatures (*e.g.*, 575 °C at 1 h hold compared to 650 °C with no dwell time). To probe this further, we investigated the effect of different dwell times at 500 °C. The data (Figure 9b) show that it is in fact possible to achieve an almost Rietveld-pure sample of Fe_3_C at 500 °C (Table 2), which is substantially lower than has previously been found in sol–gel synthesis. As in the previous cases, the Fe_3_C phase begins to form while FeO*_x_* is still present, demonstrating the stability of the Fe_3_C phase and the challenge in isolating Rietveld-pure Fe_3_N. While apparently phase-pure samples of Fe_3_N have been prepared previously from sol–gel synthesis, Rietveld refinements were not performed, and so it is likely that there were minor iron oxide and carbide contributions that were not accounted for.^15^

**Table 2:** Weight Percentage Compositions for Samples Heated at 5 °C min^–1^ to 500 °C with Various Dwell Times. Errors are Shown in Brackets

	Fe_3_O_4_	FeO	Fe_3_N	Fe_3_C
0 h	60(18)		40(18)	
0.5 h	60(3)	8(3)	31(2)	
1 h	60(2)		40(2)	
2 h	44(5)		41(5)	15(3)
3 h	27(5)	31(10)	37(6)	4.1(18)
6 h			4.4(7)	95.6(7)

## 4 Conclusions

*In situ* WAXS and SAXS studies have been used to probe the evolution of FeO*_x_*, Fe_3_N, and Fe_3_C nanoparticles from a Fe(NO_3_)_3_/gelatin sol–gel precursor. We have demonstrated that the oxide–nitride–carbide transformation happens over a range of timescales, meaning some particles transform quickly to carbide while others remain in an oxide phase until much higher temperatures. It is proposed that this is due to the nanoparticles of the oxide intermediate being of varying size, which affects the rate of carbothermal reduction and nitridation. While the oxide particle size distribution is fairly small, this broadens rapidly during the oxide–nitride phase transition, indicating that the nitride particle form by mass transport of iron from several adjacent iron oxide particles. The *in situ* WAXS data show convincing evidence for the presence of an iron carbonitride intermediate. This suggests that the iron nitride-to-carbide transformation occurs by diffusion of carbon atoms into the nitride particles. Finally, we demonstrate that while it is very difficult to isolate a phase-pure sample of Fe_3_N via this sol–gel method, Rietveld-pure Fe_3_C can be produced at the remarkably low temperature of 500 °C with a long furnace dwell time. While we have only studied the Fe–N–C system, many other transitions metals can also form nitrides and carbides so these observations may also provide helpful insight into those systems.

## Supporting Information Available

Additional experimental information, SAXS data with fits, additional particle size histograms, *ex situ* XRD data, and crystalline compositions based on Rietveld refinement (PDF)
