# Supplementary material for: In Situ and Ex Situ X-ray Diffraction and Small-Angle X-ray Scattering Investigations of the Sol–Gel Synthesis of Fe3N and Fe3C
Source: Inorg Chem. 2022 Apr 26;61(18):6742–9. doi: 10.1021/acs.inorgchem.1c03442 (PMC9092341; doi:10.1021/acs.inorgchem.1c03442)
Supplement: Supplementary file 1 — ic1c03442_si_001.pdf [file ic1c03442_si_001.pdf]

Supporting Information

In Situ and *Ex Situ* X-ray Diffraction and Small-Angle X-ray Scattering Investigations of the Sol-Gel Synthesis of Fe_3N and Fe_3C: Supplementary Information

Matthew S. Chambers,*[1] Robert D. Hunter,[1] Martin J. Hollamby,[2] Brian R. Pauw,[3] Andrew J. Smith,[4] Tim Snow,[4] Ashleigh E. Danks[1] and Zoe Schnepp*[1]

1) School of Chemistry, University of Birmingham, Birmingham, B152TT, UK, E-mail: m.s.chambers@bham.ac.uk, z.schnepp@bham.ac.uk
2) Department of Chemistry, School of Chemical and Physical Sciences, Keele University, Staffordshire, ST55BG, UK
3) Bundesanstalt für Materialforschung und -prüfung (BAM), Unter den Eichen 87, Berlin 12205, Germany.
4) Diamond Light Source, Didcot, Oxfordshire, OX11 0DE

Supplementary Experimental Information

SAXS data was processed using the DAWN software package[1,2] with the following processing steps in order: masking, correction for counting time, dark-current, transmission, primary beam flux, background (no sample in the beam), detector efficiency, flat-field, and solid angle, followed by a thickness correction and azimuthal averaging. The apparent sample thickness was estimated by using the sample absorption, composition and gravimetric density, allowing the data to be scaled to absolute units. Photon counting uncertainties were propagated through the correction steps, and the merging of datasets from the various distances is performed weighted by the datapoint uncertainty for improved resultant statistics.

Supplementary results

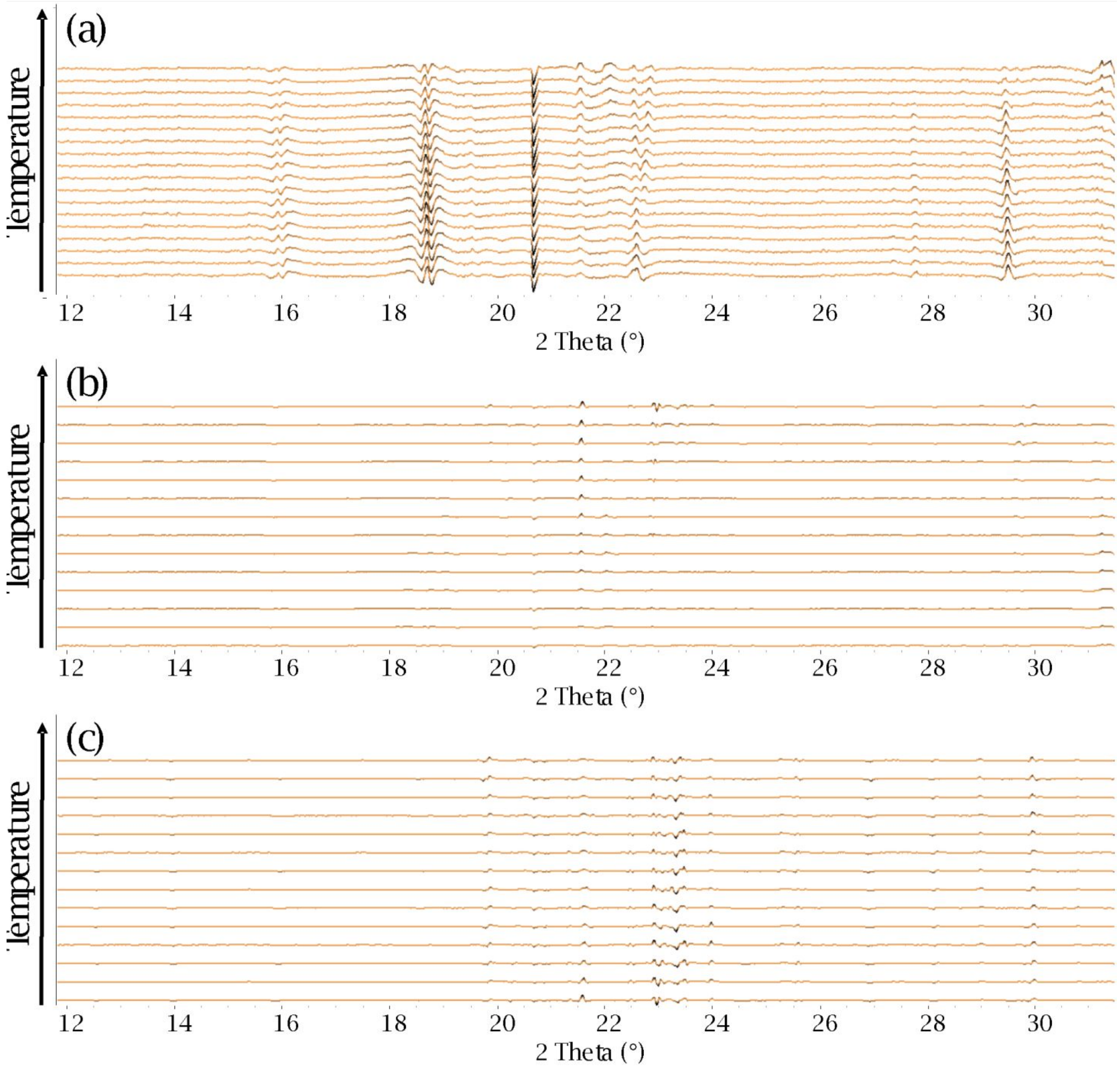

Figure S1 Difference between the observed data and calculated fit from Rietveld refinement of *in situ* synchrotron WAXS data for gelatin/$Fe(NO_3)_3$ from a) 500 – 600 °C, b) 600 – 675 °C and c) 675 – 700 °C.

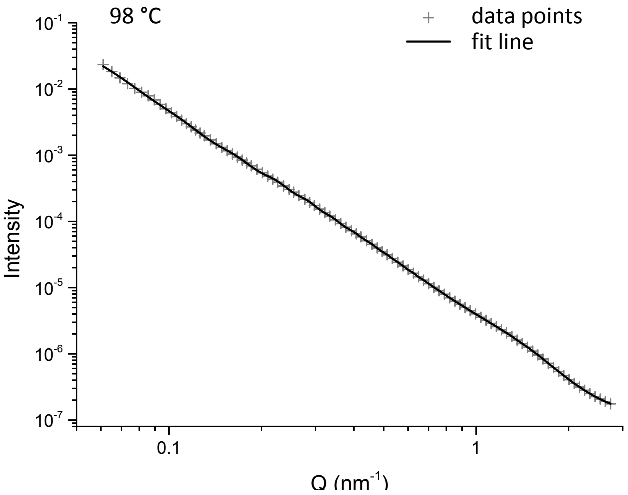

Figure S2 *In situ* SAXS data and fit line for sample at 98 °C

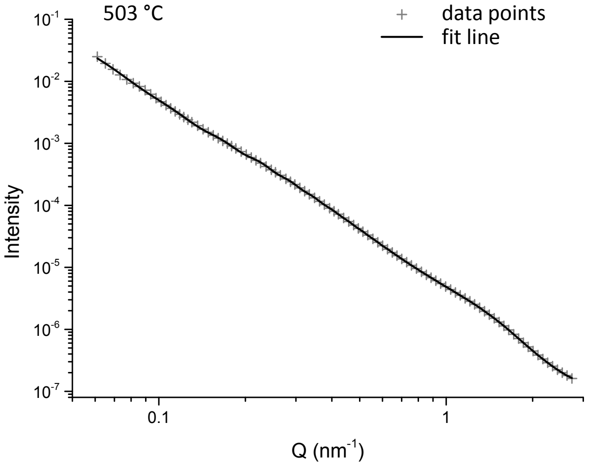

Figure S3 *In situ* SAXS data and fit line for sample at 503 °C

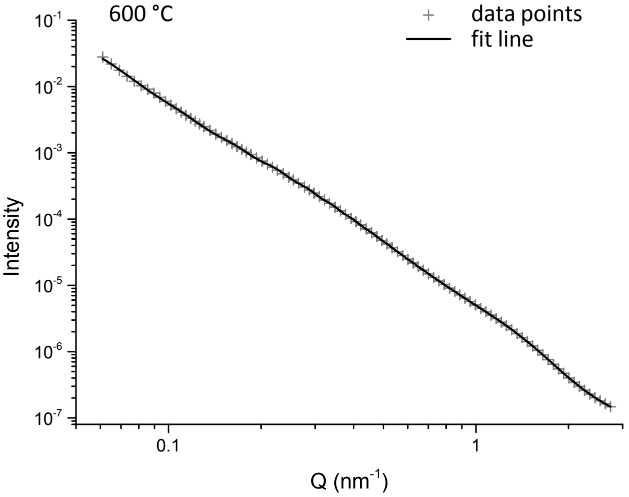

Figure S4 *In situ* SAXS data and fit line for sample at 600 °C

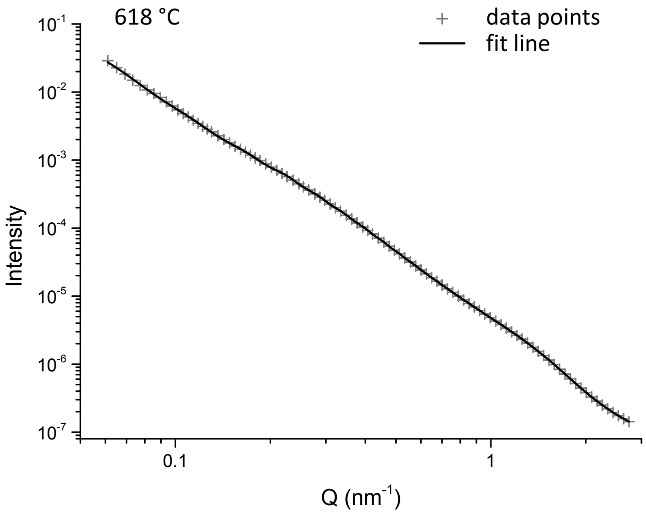

Figure S5 *In situ* SAXS data and fit line for sample at 618 °C

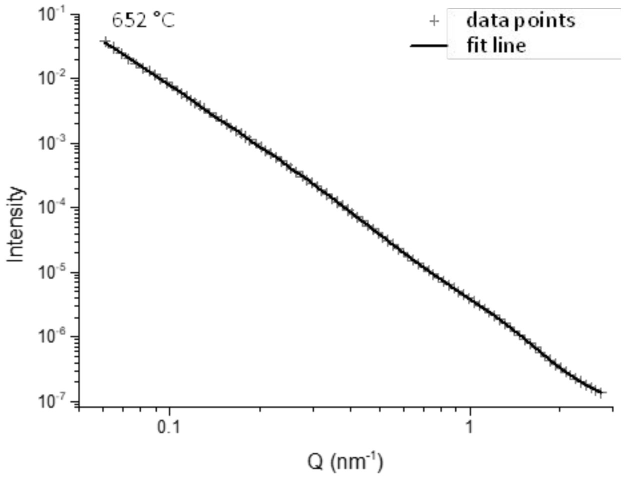

Figure S6 *In situ* SAXS data and fit line for sample at 652 °C

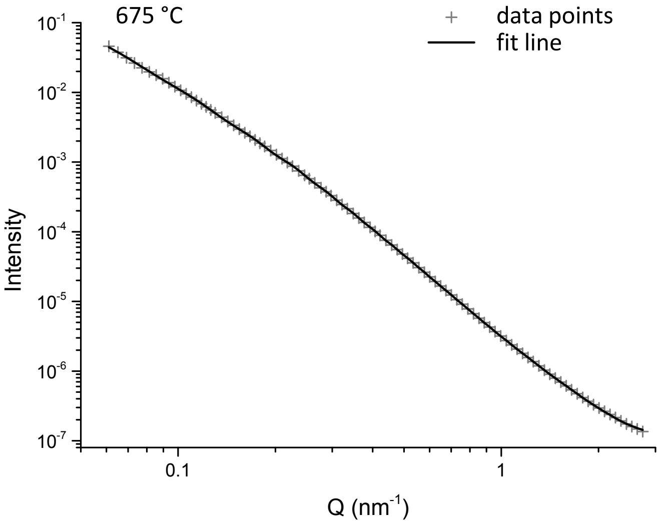

Figure S7 *In situ* SAXS data and fit line for sample at 675 °C

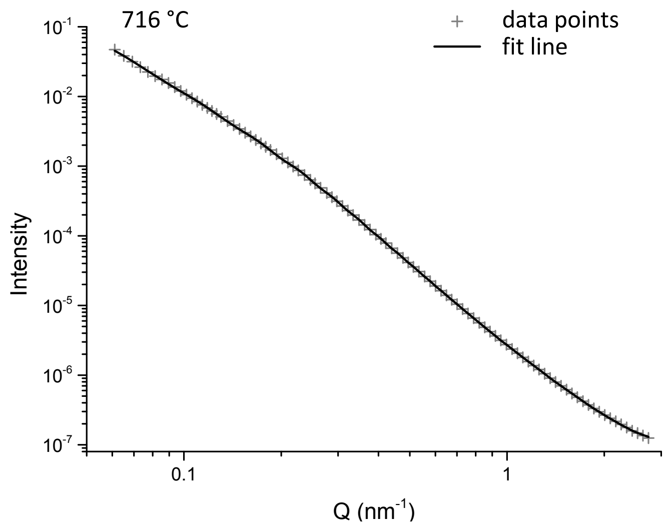

Figure S8 *In situ* SAXS data and fit line for sample at 716 °C

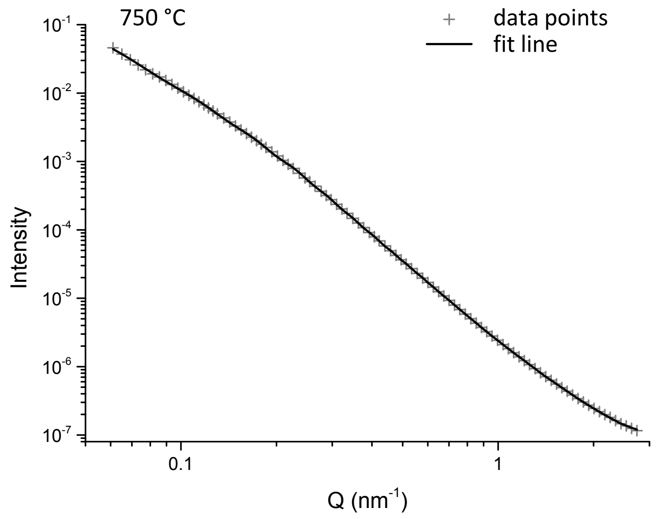

Figure S9 *In situ* SAXS data and fit line for sample at 750 °C

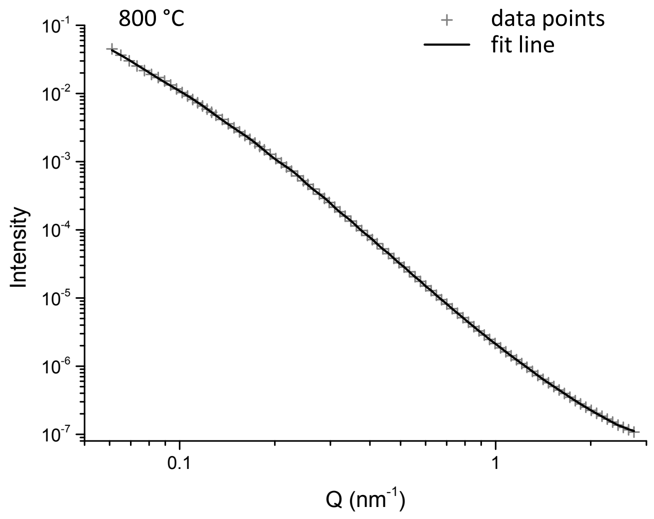

Figure S10 *In situ* SAXS data and fit line for sample at 800 °C

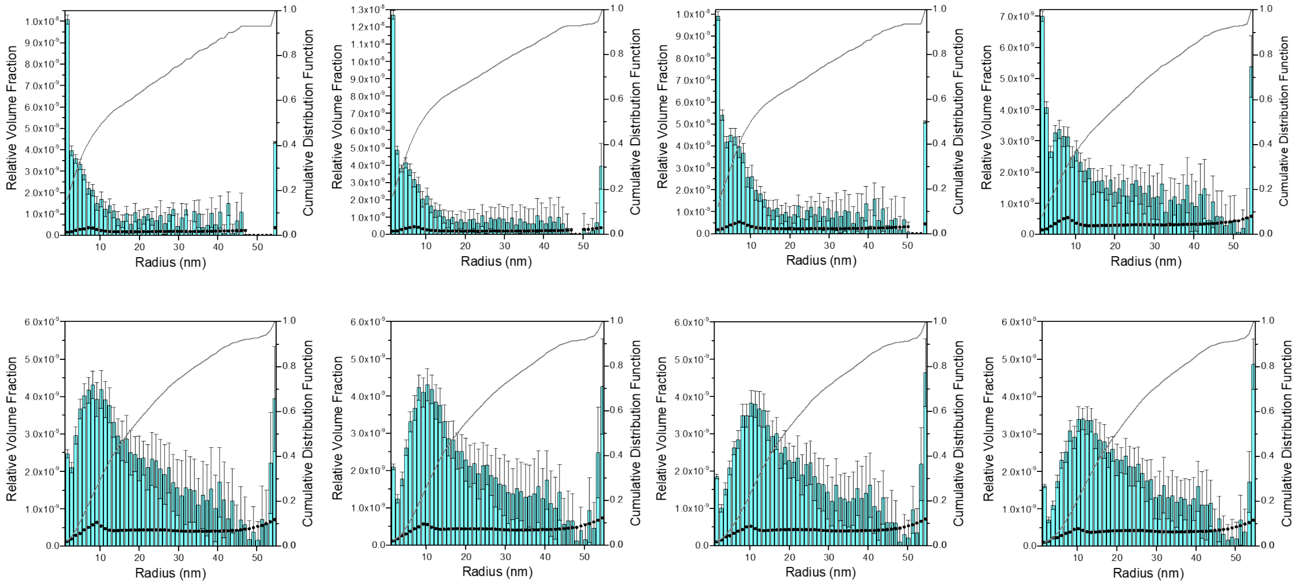

Figure S11 Particle size histograms derived from the SAXS data at a) 98 °C, b) 503 °C, c) 618 °C, d) 652 °C, e) 675 °C, f) 716 °C, g) 750 °C and h) 800 °C, with blue bars showing relative volume fraction, black dots showing minimum visibility limit and grey lines showing the cumulative distribution function.

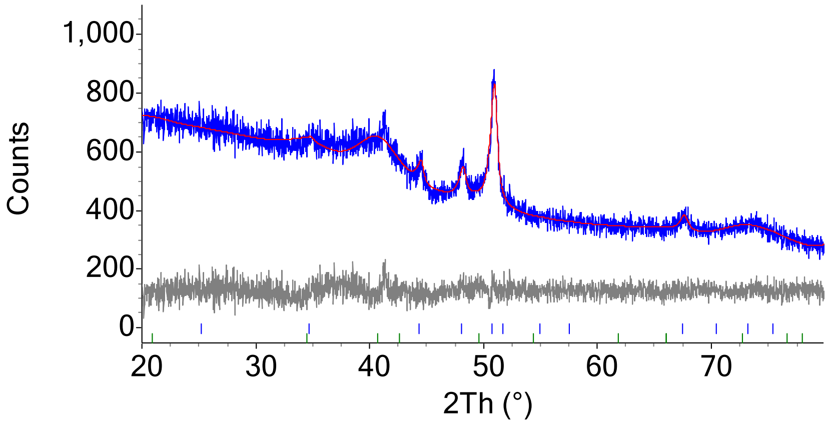

Figure S12 XRD data and Rietveld plot obtained *ex situ* from the sol-gel synthesis of $Fe(NO_3)_3$/gelatin system with a heat rate of 5 °C min^{-1} and a maximum temperature of 560 °C. The blue curve represents the observed data, the red curve the calculated pattern and the grey curve the difference between the two patterns. The tick marks represent different reference peaks, including Fe_3O_4 (green) and Fe_3N (blue).

Table S1 Composition data for samples heated to various temperatures with a hold time of 0.5 hours.

	Fe_3O_4	FeO	Fe_3N	Fe_3C
**500 °C**	60(3)	8(3)	31(2)	
**520 °C**	49(10)		31(7)	20(11)
**540 °C**	28(10)		25(4)	47(7)
**560 °C**	44(8)		29(4)	27(5)

Table S2 Composition data for samples heated to various temperatures with a hold time of 1 hours.

	Fe_3O_4	Fe_3N	Fe_3C
**500 °C**	60(3)	40(2)	
**520 °C**	34(10)	29(5)	38(7)
**550 °C**		4.2(5)	95.8(5)
**575 °C**			100%

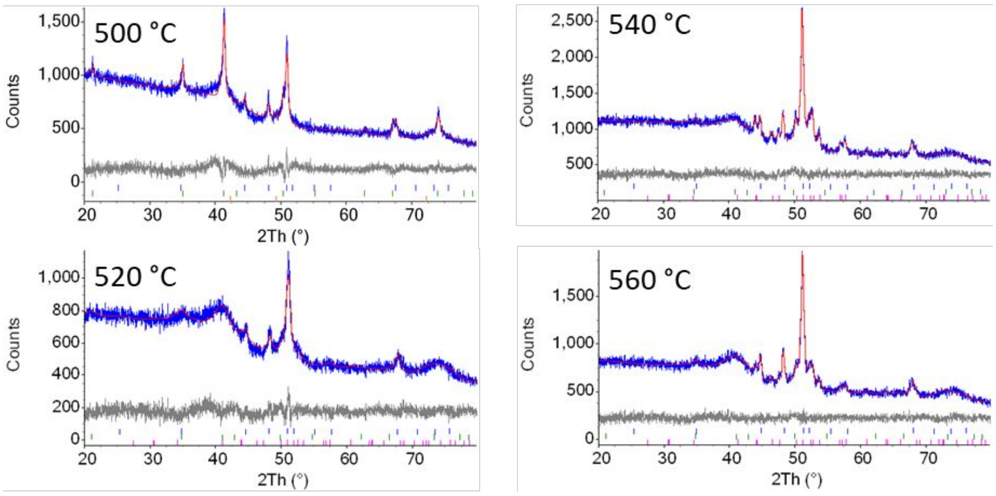

Figure S13 - XRD data and Rietveld plots obtained *ex situ* from the sol-gel synthesis of $Fe(NO_3)_3$/gelatin system with a heat rate of 5 °C min^{-1} and 0.5 h dwell time at various temperatures. The blue curves represent the observed data, the red curves the calculated pattern and the grey curves the difference between the two patterns. The tick marks represent different reference peaks, including Fe_3O_4 (green), FeO_x (orange), Fe_3N (blue) and Fe_3C (pink).

Supporting Information References

[1] Filik, J.; Ashton, A. W.; Chang, P. C. Y.; Chater, P. A.; Day, S. J.; Drakopoulos, M.; Gerring, M. W.; Hart, M. L.; Magdysyuk, O. V.; Michalik, S.; Smith, A.; Tang, C. C.; Terrill, N. J.; Wharmby, M. T.; Wilhelm, H., Processing two-dimensional X-ray diffraction and small-angle scattering data in DAWN 2. *J. Appl. Crystallogr.*, **2017**, 50, 959–966.

[2] Pauw, B. R.; Smith, A. J.; Snow, T.; Terrill, N. J.; Thünemann, A. F.; The modular small-angle X-ray scattering data correction sequence. *J. Appl. Crystallogr.*, **2017**, 50, 1800–1811.
